# Towards engineering and production of artificial spider silk using tools of synthetic biology

**DOI:** 10.1049/enb.2019.0017

**Published:** 2020-03-16

**Authors:** Hashwardhan Poddar, Rainer Breitling, Eriko Takano

**Affiliations:** ^1^ Faculty of Science and Engineering, Manchester Institute of Biotechnology, Manchester Synthetic Biology Research Centre SYNBIOCHEM The University of Manchester Manchester M1 7DN UK

**Keywords:** biomimetics, biomedical materials, proteins, molecular biophysics, bio‐inspired materials, biochemistry, reviews, artificial spider silk, synthetic biology, spider silk proteins, synthetic spider silk

## Abstract

Spider silk is one of the strongest biomaterials available in nature. Its mechanical properties make it a good candidate for applications in various fields ranging from protective armour to bandages for wound dressing to coatings for medical implants. Spider silk is formed by an intricate arrangement of spidroins, which are extremely large proteins containing long stretches of repeating segments rich in alanine and glycine. A large amount of research has been directed towards harnessing the spectacular potential of spider silks and using them for different applications. The interdisciplinary approach of synthetic biology is an ideal tool to study these spider silk proteins and work towards the engineering and production of synthetic spider silk. This review aims to highlight the recent progress that has been made in the study of spider silk proteins using different branches of synthetic biology. Here, the authors discuss the different computational approaches, directed evolution techniques and various expression platforms that have been tested for the successful production of spider silk. Future challenges facing the field and possible solutions offered by synthetic biology are also discussed.

## 1 Introduction

Spider silk has fascinated and surprised humans since ancient times with its unique mechanical properties, which combine strength, toughness and elasticity [1, 2] in a material superficially notable for its delicacy and fragility [3, 4]. The remarkable properties of spider silk have been characterised and well documented and many applications have been suggested. Spider silk has been found to be five times stronger than steel (by weight) and three times stronger compared to Kevlar [5, 6]. Spider silk has been proposed as a suitable material for protective armour [7], as well as fashionable clothing and artwork [8, 9]. It can also be used to make parachute cord and industrial cables [10] and also in the production of composite materials to be used in the aviation industry [10, 11]. In addition to their mechanical versatility, spider silk proteins are soluble in water and biodegradable. This allows spider silk to be used as biodegradable bandages in the treatment of wounds [10, 12], as a carrier for drug delivery [11, 13] and also as scaffolds for growing cells and tissues [10, 11, 12, 13]. Spider silk's biocompatibility also makes it a promising material to employ in coatings for medical implants [14]. Owing to its enormous potential in a variety of different applications, intense research efforts have been directed towards the study of different spider silk proteins from various species.

## 2 Spider silk proteins

Spiders produce different types of silk for specific purposes, with up to seven different silk types found in a single species (Table 1, [15, 16, 17, 18]). All of them are produced in specialised abdominal glands.

**Table 1:** Different spider silks and their predominant uses

Silk types	Use
Major ampullate silk	Dragline silk – used to make the framework of the web and also as a safety line during falls. Strongest of the spider silk varieties.
Minor ampullate silk	Used to make the auxiliary spiral of the web for reinforcement.
Flagelliform silk	Found in the core fibres of the capture spiral.
Tubuliform silk	Forms the outer protective layer of the egg sac.
Aciniform silk	Provides soft inner lining of egg sac and also wrapping for freshly captured prey.
Aggregate silk	Forms the sticky aqueous coating of the capture spiral and has adhesive properties.
Pyriform silk	Functions as cement between separate threads and also for attachments to construct a stable web.
Cribellar silk	Forms the dry adhesive component of the prey‐catching silk of cribellate spiders.

**Fig. 1:**
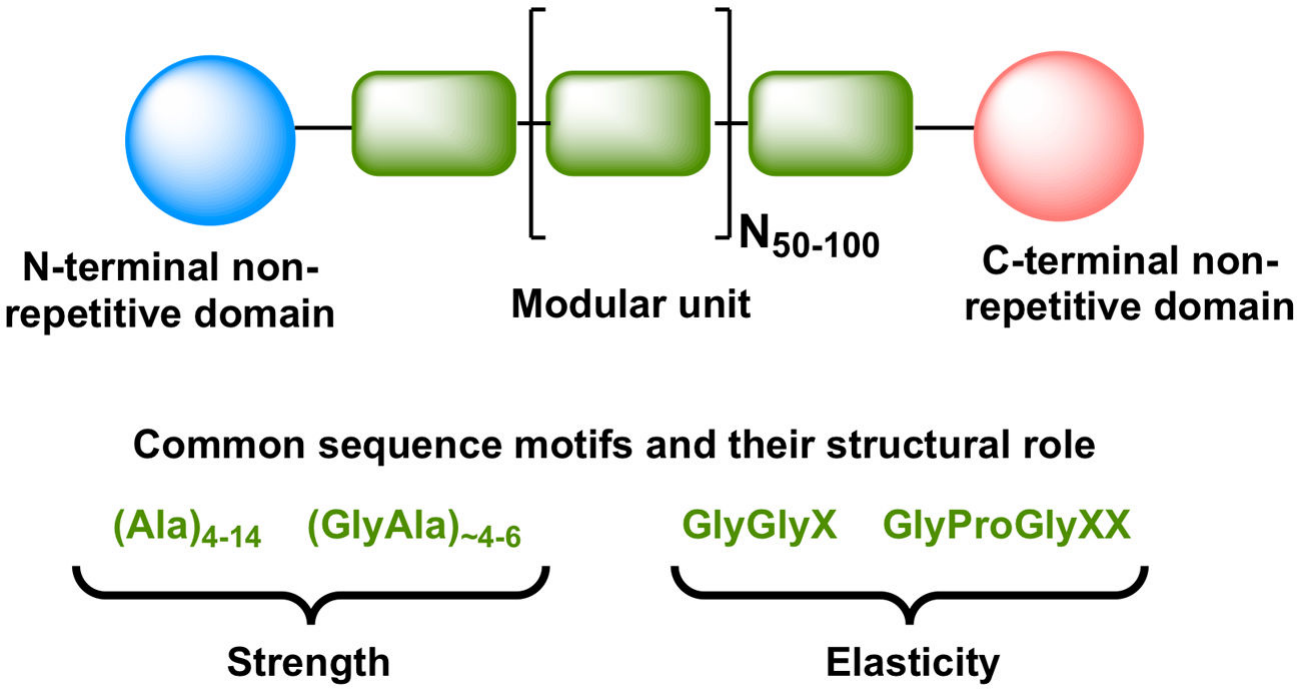
Modular structure of a spidroin protein depicting the globular N‐ and C‐terminal domains in blue and pink, respectively. The repetitive modular units are shown in green

Spider silk proteins are commonly referred to as spidroins. Spidroins are typically 250–350 kDa in size and have a largely conserved architecture [19], consisting of three distinct regions – a conserved, non‐repetitive and globular N‐terminal (∼130 residues) [20] and C‐terminal domain (∼110 residues) (Fig. 1, [21]), which flank the highly repetitive alanine and glycine‐rich region. The number of spidroins found in a single species of spider is typically even larger than the number of different major silk types, but the spidroin terminal domains are evolutionarily conserved across spider species and silk types and have been well characterised [17, 20, 21, 22]. They each form a four‐ or (more commonly) five‐helix bundle and have been shown to play crucial roles in silk fibre assembly by responding to changes in the pH and ionic concentration in the silk glands during the spinning process [17, 21, 22, 23, 24, 25]. The repetitive region, in contrast, shows more variability between different silk types and even within the same spidroin class from different species [26]. A typical spidroin can contain hundreds of repeats of a particular modular unit (40–200 amino acids) and can form up to ∼90% of the total amino acids of the spider silk protein [27, 28]. The modular units often include the following sequence motifs: (Ala)_4−14_, (GlyAla)_∼4−6_, GlyGlyX and GlyProGlyXX (where X represents a variable amino acid residue) [17, 27]. These motifs are known to form particular secondary structure motifs in spider silk. There is a direct correlation between the composition of these modular units and the mechanical properties exhibited by the silk fibre. For instance, the presence of long poly‐alanine segments makes the fibres strong, whereas glycine‐rich stretches are known to impart flexibility and make the fibres more extendible [29]. Interestingly, a direct relationship between the size of the proteins and their mechanical properties has not been established yet [30], which implies that shorter spidroins containing fewer repetitive regions can also be used for synthetic spider silk production [17]. Indeed, Rising and co‐workers have successfully shown that recombinant expression of a chimeric minispidroin, containing only a relatively short repetitive region, can yield synthetic silk, which is comparable to natural spider silk in its mechanical properties [31].

## 3 Synthetic biology – a versatile toolkit

Synthetic biology can be defined as an interdisciplinary branch of biology, which uses the principles of engineering to design and generate new biological entities with useful properties [32]. It encompasses techniques and principles from various research fields that include molecular biology, computer science/bioinformatics, statistics, biochemistry and engineering [33, 34, 35]. There have been many examples of using synthetic biology to engineer biological systems, for example for the efficient production of chemicals, healthcare products or biomaterials [36, 37, 38, 39, 40]. The growth in the field of synthetic biology has been largely fuelled by the rapidly declining costs of both DNA sequencing and synthesis [41]. In recent years, a lot of interest has been generated in using synthetic biology to produce novel bio‐based materials, with spider silk being a popular target [42, 43, 44]. In the following sections, we discuss how different techniques in our synthetic biology toolkit can be used to efficiently produce synthetic spider silk.

## 4 Computational and bioinformatics techniques

The first step in the process of production of synthetic spider silk is to identify novel spider silk sequences. A number of bioinformatics tools have been developed to analyse and identify amino acid repeats in fibrous proteins, including spider silk [45, 46]. Good knowledge of spider sequences from different silk types and species is necessary to establish a link between the evolution of different spider species and the corresponding mechanical properties of their silk fibres. Recently, Jung *et al.* devised a novel *in silico* approach to analyse the imperfect repeats in groups of spider silk sequences [47]. This approach helped them to identify frequently appearing trimer motifs and conserved sequence patterns from different sequences, which allowed them to establish a direct link between the evolutionary emergence of major spider groups, the appearance of new repeat motifs, and the mechanical properties of the associated silk fibres.

Another method to find novel silk sequence motifs focuses on transcriptome data, instead of protein sequences. Malay *et al.* queried the NCBI Sequence Read Archive [48] to find transcripts resembling MaSp (dragline silk) sequences in an expanded range of species [49]. Their analysis revealed a number of different conserved repeat motifs, which they tentatively link to specific biological functions. Most importantly, they identify patterns of correlation between motif abundance and spider web architecture (and the associated prey capture strategies), indicating the importance of individual motif composition, rather than total repeat length, in modulating the mechanical properties of the silk fibres.

Kono *et al.* present another fine example of how a more focused database can give valuable information [50]. In their study, a specific catalogue containing the full set of spidroin genes for the orb‐weaving spider *Araneus ventricosus* was carefully curated, identifying several novel sequences. Importantly, they report the first full‐length sequence MaSp3, a new paralog of dragline silk spidroin. This expansion of the dragline spidroin complement beyond the usual components, MaSp1 and MaSp2, appears to be conserved only in a subset of orb‐weaving spiders [51], but is an evolutionary novelty shared by other members of the Araneoidea, in particular the tangle‐web spiders of the family Theridiidae [52]. The increasing availability of complete spider genome sequences and in‐depth transcriptome datasets of various spider tissues is providing an abundance of new insights into the evolution of the mechanical diversity of spider silk, but also reveals new potential functions for these proteins. Several studies have found non‐gland‐specific expression of some spidroins [53, 54], and most interestingly spidroins can be found highly expressed in venom glands [19], although it is not yet clear how prevalent this phenomenon is and whether the venom‐gland derived silk component of the prey‐catching silk of spitting spiders (Scytodidae) is homologous to the abdominally expressed regular silk types [55, 56]. On the other hand, some silk types have been shown to contain a surprising diversity of non‐spidroin proteins [57], possibly with important functions in modulating the mechanical and biological properties of native silk. Future studies of this rich diversity might yield important insights into the optimal strategy for producing biomimetic spider silk by a heterologous expression, which currently employs only single spidroin proteins, rather than complex mixtures.

## 5 Directed evolution techniques

Spiders have evolved their spidroin sequences over millions of years to perform specific functions. This has resulted in highly optimised proteins for these native functions – but not necessarily the ideal sequences for heterologous expression and the diverse range of non‐natural applications envisaged in synthetic biology. To optimise natural template proteins for specific applications, directed evolution has emerged as a key tool, as recognised by the 2018 Nobel Prize for Chemistry [58, 59]. The modular nature of spidroins and the clear correlation between sequence composition and mechanical properties makes them an interesting target for directed evolution strategies.

The first step in the process of directed evolution is the design and synthesis of desired gene segments, which can be derived from a natural source (e.g. known spidroin sequences) or guided by computational predictions [60]. The most common and established protocols for the introduction of mutations are based on the technique of polymerase chain reaction (PCR). Point mutations can be introduced randomly across the sequence using error‐prone DNA polymerases or in a more targeted fashion by an assembly of DNA oligomers containing variable nucleotide sequences [59]. DNA shuffling is another widely used method, which involves digesting similar DNA sequences with DNAse I endonuclease enzymes to create random short fragments. These fragments are then re‐assembled randomly to make chimeras containing sequences from several original source sequences [61, 62]. Such an approach is ideal for creating diversified libraries of proteins containing imperfectly repetitive regions of major functional relevance, such as spidroins. Libraries of chimeric spidroins, based on sequence elements from different spider species, as well as based on consensus motifs, are likely to contain sequences representing a wide range of mechanical properties. Golden Gate assembly can be used for a more targeted variation of DNA shuffling; it entails digestion of different sequences with type‐II restriction enzymes, followed by *specific* reassembly of modular DNA in a single pot [63]. This approach allows for a more even coverage of the design space than would typically result from a random re‐assembly, which is often biased towards specific types of constructs. For all of these methods, the resulting collections of sequences can be introduced into a relevant expression vector and transformed into the host organism for their recombinant expression and subsequent functional screening. In a full synthetic biology approach, this is then followed by the learning of general design rules followed by further rounds of library construction, focusing on the most successful designs for a particular application.

Directed evolution techniques also offer opportunities to engineer spider silk proteins for desired properties. An example of how introducing mutations in the repetitive region of the spidroin can lead to altered mechanical characteristics was presented by Grip *et al.* [64]. It was shown that replacing two alanine residues in the first repetitive segment of a minispidroin (comprising a total of four segments) with cysteine residues improved the tensile strength and stiffness of the resulting fibre; whereas similar mutations in the fourth poly‐Ala block resulted in premature aggregation of the protein, possibly due to the formation of disulphide bonds with a conserved cysteine residue in the C‐terminal domain. Directed evolution of a target can be immensely aided by the knowledge of its structure. Solving crystal structures of the individual terminal domains will help in explaining the underlying mechanism of dimerisation during the spinning process and also the role of different amino acid residues. Crystal structures of the N‐terminal domain from *Nephila clavipes* (Araneidae) and *Euprosthenops australis* (Pisauridae) have been solved, and these structures have shed light onto the details of the molecular events occurring during the pH‐induced dimerisation of these domains [23, 24, 25]. More importantly, these structures allowed the identification of key residues, which are crucial for dimer formation. Obtaining similar molecular structure data for the C‐terminal domain and, if possible, the minispidroin or even intact native spidroins would go a long way in expanding our understanding of fibre formation.

## 6 Different host systems for spidroin production

Perhaps the biggest challenge currently facing the efficient production of spider silk proteins is finding the right host for expression in large quantities. This is not only limiting the industrial production of biomimetic spider silk, but also the efficient screening of engineered silk libraries for a novel or improved properties. In contrast to silkworms, which were domesticated already in ancient times [65], the territorial, carnivorous and often cannibalistic nature of most spider species has been a major impediment to farming spiders for their silk [66, 67]. Thus, to harness spider silk for biotechnological purposes, an efficient heterologous production system is needed, and over the years, several platforms offered by synthetic biology have been tested for the production of spidroins ([68], Table 2).

**Table 2:** Different platforms investigated for recombinant spider silk production, based on major ampullate spidroins

Platform category	Platform	Protein size, kDa	Comments	Reference
Bacteria	*Escherichia coli*	native‐sized spidroin of 284.9	500–2700 mg/l of spidroins produced. Spiber Technologies AB, involved in the production of synthetic spider silk, also used *E. coli* as a platform.	[30, 69, 70, 71, 72]
Bacteria	*Salmonella typhimurium*	30–56	Used to secrete spidroin into the growth medium to bypass the complex purification process.	[73]
Yeast	*Pichia pastoris*	65	∼663 mg/l spider silk protein obtained. Bolt Threads, another commercial enterprise, uses yeast as their platform.	[74, 75]
Plant	Tobacco (*Nicotiana tabacum*) and potato (*Solanum tuberosum*)	13–100	Spidroin produced up to 0.5% of total proteins.	[76]
Mammalian cells	Bovine mammary epithelial and baby hamster kidney mammalian cells	60–140	∼25–50 mg/l of spider silk protein produced in BHK cells.	[77]
Mammal	Transgenic mice and goats	31–66	11.7 mg/l of spider silk produced.	[78, 79]
Insect	*Bombyx mori*	70–130	Chimeric proteins with spider silk content of up to 35.2% (w/w) of cocoon shells. Kraig Biocraft Laboratories have also successfully used silkworms for the production of synthetic spider silk.	[80, 81, 82, 83]

Baker's yeast and *Escherichia coli* were the first organisms to be used for recombinant expression of spidroin proteins [69, 74]. These unicellular species are genetically tractable and cost‐effective to grow at industrial scales. In particular, *E. coli* has evolved into a workhorse for recombinant protein production [84]; however, initially, low‐level expression of spidroins was reported and the largest spidroin that could be produced was only 43 kDa in size [70]. This inefficiency was attributed to the highly repetitive nature of the amino acid sequence of spidroins, which led to DNA deletion in the spidroin gene and errors during the transcription and translation processes [69]. Furthermore, it was reported that the alanyl‐ and glycyl‐tRNA levels in silk glands are increased prior to translation to satisfy the high demand of alanine and glycine [85]. This phenomenon does not occur readily in *E. coli* cells during the expression of proteins. Xia *et al.* were the first to overcome these challenges and report the successful production of native‐sized (284.9 kDa) spidroin in *E. coli* [30], with improved mechanical performance. More recently, a 557 kDa spidroin was produced as a recombinant protein in *E. coli*. The production of this large protein was made possible by expressing two native‐sized spidroins, which were subsequently fused using inteins [71]. Engineered *Salmonella typhimurium* was used to produce and export silk proteins into the growth medium, exploiting the type III secretion system to eliminate the need for a complex purification step in the production of spidroins [73].

Mammalian cell lines have also been tried as a possible expression system for spidroins [77], making use of their ability to more efficiently produce larger eukaryotic proteins when compared to bacterial or yeast systems. The effect of post‐translational modifications of spider silk proteins on fibre formation and its mechanical properties needs to be investigated in detail, but it has already been shown that both MaSp1 and MaSp2 from *Nephila clavipes* dragline silk fibres have phosphorylation sites in the repetitive regions [86, 87]. It has been reported that phosphorylation increases the solubility of recombinantly expressed spidroin, whereas dephosphorylation promotes aggregation [88]. A direct correlation, however, between phosphorylation of the native spider silk proteins and their solubility and expression levels in mammalian and microbial hosts needs to be established and further investigated [89].

Several multicellular organisms, such as transgenic plants and animals, have also tested as suitable platforms for the expression of recombinant spidroin proteins [60]. Notably, transgenic mice and goats have been investigated for their ability to produce and secrete spidroins through their mammary glands [78, 79]. However, these expression hosts require further studies and development as only relatively small quantities of spidroins were obtained. The obvious multicellular platform that suggests itself for the large‐scale production of spider silk would be the silkworm (*Bombyx mori*), and the necessary genetic engineering techniques have become available in recent years. Silkworms are known to produce large amounts of silk themselves and efficiently spin them into fibres, thus making them immensely attractive hosts for recombinant expression of spidroins [90]. In the ideal scenario, the recombinant silk produced by silkworms would be in a near‐finished quality, and would not require any further processing by various non‐natural or biomimetic spinning methods [17, 31, 66]. Currently, there are still some limitations to using silkworms as a platform, for spider silk production. The high insolubility of the spidroins and the inability of the silkworms to spin spidroins into fibres are major obstacles [91]. To overcome this challenge, the tools of synthetic biology were used to create transgenic silkworms that express silkworm/spider chimeric spidroins, in which the repetitive regions responsible for the elasticity and strength of spider silk were flanked by the N‐ and C‐terminal domains of silkworm silk [80]. The resulting chimeric fibres, spun by these engineered animals, were shown to achieve the same toughness as native spider silk, outperforming the parental silkworm silk. An alternative approach employed another ingredient of the synthetic biology toolbox, transcription activator‐like effector nuclease (TALEN)‐mediated homology‐directed repair, to replace one of the native silkworm silk genes by a spidroin gene from *Nephila clavipes*, under the control of the native silkworm promoter [81]. The resulting composite silk, which contained up to 35.2% of spidroin, showed increased extensibility, but inferior strength, compared to native silkworm silk. More recently, a CRISPR/Cas9 based strategy was successfully used to incorporate spider silk genes in the silkworm genome [92]. Progress in achieving more spider‐like mechanical performance will probably depend on the more extensive exploration of alternative spidroin sequences, including different repeat patterns and larger repetitive regions.

## 7 Future perspectives and challenges

Synthetic biology has enormous potential to offer sustainable and green solutions for the production of a number of commodities, including biomaterials. The study of spider silks can immensely benefit from the progress of synthetic biology, and several of the recent advances in recombinant spider silk production – ranging from the identification of evolutionary design patterns correlating with mechanical properties to the engineering of silkworms for the production of mechanically enhanced chimeric silks – have only been possible due to the power of this cross‐disciplinary approach.

A big obstacle in the high‐throughput screening of spider silk variants is the necessity to characterise the mechanical properties of these new variants to determine their efficiency. The libraries of variants can be generated relatively rapidly by using automation and liquid handling robots, but purification and processing of the expressed spidroin variants in a high‐throughput mode is still a big challenge. Furthermore, substantial amounts of protein are needed to perform the mechanical characterisation, which further acts as an impediment in this process. It has been suggested that protocols to measure alternate factors, which directly correlate with the desired mechanical property, in a high‐throughput setting, have to be established [93]. This approach could help narrow down the pool of variants (hundreds or even thousands), so that only a selected few covering the spectrum of desired properties can be taken to the next stage of large‐scale material tests and subsequent learning of design rules. Molecular modelling and simulation constitute another approach that can be used to further streamline the synthetic biology process. Rim *et al.* proposed an iterative protocol in which multiscale modelling is used to predict silk fibre properties based on the protein composition [94]. Model predictions are validated by carrying out DNA synthesis, protein expression, and subsequent mechanical testing. The data gathered is used to improve the predictive rules and thus feeds back into the iterative process.

Another challenge facing the successful production of artificial spider silk is the development of techniques for efficient spinning of spider silk, which can rival natural spider silk in its mechanical properties. Although a lot of research has been dedicated to understanding the events in the silk glands and the spinneret during spinning, translating them in vitro remains a challenge. Most importantly, obtaining feedstock dopes in high concentrations of up to 30–50% (w/w) remains difficult, and mimicking the apparatus of a spider's spinneret is also challenging [17]. Several techniques that have been developed include electrospinning [95, 96], spidroin self‐assembly at air‐water interfaces [97, 98], and the use of microfluidic devices [99]. It is to be noted here that none of these techniques have managed to match the mechanical properties of naturally produced spider silk. Koeppel and Holland, in their recent review, have discussed all available techniques in detail and also highlighted the challenges facing this important aspect of artificial spider silk production [66].

Overcoming all the challenges described above is important, but for the large‐scale commercial production of spider silk to be viable, it needs to be cost efficient. Currently, several companies are involved in the production of spider silk using different methods. Kraig Biocraft Laboratories, using their genetically modified silkworms [80], have successfully managed to produce a kilogram of spider silk for ∼300 USD. This is by far the most economical spider silk production reported so far [82], but further improvements are needed to bring down the costs for the economical production of artificial spider silk on a large scale. Both, synthetic biology and spider silk research are currently undergoing a phase of rapid progress – combining their forces will result in the rapid realisation of the full potential of spider silk as a key ingredient to a broad range of valuable biomimetic materials.
